# *In Utero* and Lactational Exposures to Low Doses of Polybrominated Diphenyl Ether-47 Alter the Reproductive System and Thyroid Gland of Female Rat Offspring

**DOI:** 10.1289/ehp.10536

**Published:** 2007-12-03

**Authors:** Chris E. Talsness, Sergio N. Kuriyama, Anja Sterner-Kock, Petra Schnitker, Simone Wichert Grande, Mehdi Shakibaei, Anderson Andrade, Konstanze Grote, Ibrahim Chahoud

**Affiliations:** 1 Department of Toxicology, Institute of Clinical Pharmacology and Toxicology, Campus Benjamin Franklin, Charité University Medical School Berlin, Berlin, Germany; 2 Department of Veterinary Pathology, Freie Universität Berlin, Berlin, Germany; 3 Department of Anatomy, Ludwig-Maximilians-University Munich, Munich, Germany

**Keywords:** development, endocrine disruption, *in vivo*, PBDE-47, reproductive system, thyroid

## Abstract

**Background:**

Polybrominated diphenyl ethers (PBDEs) are capable of disrupting thyroid hormone homeostasis. PBDE-47 (2,2′,4,4′-tetrabromodiphenyl ether) is one of the most abundant congeners found in human breast adipose tissue and maternal milk samples.

**Objectives:**

We evaluated the effects of developmental exposure to low doses of PBDE-47 on the female reproductive system.

**Methods:**

Pregnant Wistar rats were administered vehicle (peanut oil) or PBDE-47 [140 or 700 μg/kg body weight (bw)] on gestation day (GD) 6, or 5 mg 6-n-propyl-2-thiouracil (PTU)/L in the drinking water from GD7 through postnatal day (PND) 21.

**Results:**

In female offspring sacrificed on PND38, there was a significant decrease in ovarian weight after exposure to PTU or 140 μg/kg PBDE-47. Alterations in folliculogenesis were apparent: we observed a decrease in tertiary follicles and serum estradiol concentrations in the offspring exposed to either PTU or 700 μg/kg PBDE-47. PTU exposure also resulted in a decrease in primordial follicles. On PND100, persistent effects on the thyroid glands included histologic and morphometric changes after exposure to either PTU or PBDE-47. No relevant changes in reproductive indices were observed after mating the exposed F_1_ females with nontreated males.

**Conclusions:**

Administration of PBDE-47 at doses relevant to human exposure led to changes in the rat female reproductive system and thyroid gland.

*In vivo*, polybrominated diphenyl ethers (PBDEs) have been shown to alter thyroid hormone homeostasis (Ellis-Hutchings et al. 2006; Fowles et al. 1994; Hallgren et al. 2001; Stoker et al. 2004; Zhou et al. 2001, 2002) and neurobehavior (Dufault et al. 2005; Eriksson et al. 2001, 2002; Kuriyama et al. 2005; Sand et al. 2004; Viberg et al. 2003, 2004, 2006), as well as to influence both male and female reproductive systems (Ceccatelli et al. 2006; Kuriyama et al. 2005; Lilienthal et al. 2006; Stoker et al. 2004, 2005; Talsness et al. 2005; Tseng et al. 2006). PBDEs are used as flame retardants in a wide variety of consumer products including plastics (e.g., computer housings, small motor appliances, textiles, furniture foam, electronic and wire insulation). Their ubiquitous use, lipophilic nature, and ability to persist in the environment has resulted in their accumulation in wildlife (Hale et al. 2001; Jacobs et al. 2002; Lindberg et al. 2004; Rice et al. 2002; Zenegg et al. 2003). In addition, PBDEs have been found in sentinel animals from different trophic levels of the North Sea food web, at increasing levels moving up the food chain (Boon et al. 2002).

Of particular concern is the presence of considerable amounts of these flame retardants in human breast milk. Swedish researchers were the first to report an alarming increase (60-fold) in PBDEs in maternal milk over a 25-year period, which was equivalent to a doubling of the concentration every 5 years (Meironyte et al. 1999). Later publications have shown that PBDE concentrations are even higher in North Americans (Johnson-Restrepo et al. 2005; Mazdai et al. 2003; Petreas et al. 2003; Schecter et al. 2003), and it appears that the concentrations in breast milk of Japanese women are somewhat lower than that found in European countries (Eslami et al. 2006; Hites 2004). The possible routes of exposure include consumption of contaminated food sources (both animal and plant), indoor air, outdoor air, ingestion of dust (Jones-Otazo et al. 2005), as well as direct dermal exposure (e.g., through contact with polyurethane-foam) (Hites 2004).

Because PBDEs have structural similarities with other halogenated aromatic pollutants, it was postulated that they may be able to induce dioxin-like toxicity. Although interaction of PBDEs with cytosolic aryl hydrocarbon receptor (AhR) has been shown (Chen et al. 2001) and high doses of commercial mixtures induce ethoxy-resorufin-*O*-deethylase activity (Zhou et al. 2001), quantitative polymerase chain reaction has indicated that up-regulation of cytochrome P450 1A1 (*CYP1A1*) by the commercial DE-71 formulation was weak compared with the dioxin-like polychlorinated biphenyl (PCB)-126 (Sanders et al. 2005). In addition, results from experiments analyzing the influence of different PBDE congeners or mixtures on various steps of the AhR signal-transduction pathway leading to CYP1A1, led to suggestions that the contribution of PBDEs to overall dioxin-like toxicity is miniscule compared with PCBs and polychlorinated dibenzo-*p*-dioxins (Chen and Bunce 2003) and that PBDEs bind, but do not activate, the AhR–AhR nuclear translocator protein–xenobiotic response element complex (Peters et al. 2006).

Gene reporter assays have indicated that some PBDE congeners and/or their metabolites can activate estrogen receptor signal-transduction pathways *in vitro* and exhibit antiestrogenic activity, which may or may not be Ah receptor mediated via increased estrogen catabolism or interference with estrogen-mediated transcription (Meerts et al. 2001). *In vivo* studies in rats have shown that exposure to PBDE-99 (2,2′,4,4′,5-pentabromodiphenyl ether) affects the regulation of estrogen target genes in the uterus (Cecatelli et al. 2006).

*In vivo* studies in rats and mice have consistently shown a reduction in thyroxine (T_4_) concentrations after exposure to PBDEs (Ellis-Hutchings et al. 2006; Fowles et al. 1994; Hallgren et al. 2001; Stoker et al. 2004; Zhou et al. 2001, 2002). Thyroid hormones influence the function of nearly all tissues via their effects on cellular metabolism and the essential roles they play in differentiation and growth. Interference with thyroid hormone homeostasis by environmental compounds therefore has the potential to affect every system in the body and to impact development. Thyroid hormone is known to influence or modulate estrogen action in various species, including timing of seasonal reproduction and lordosis behavior in rodents (Vasudevan et al. 2002). In addition, late uterine responses to estradiol administration have been shown to be diminished in hypothyroid rats (Gardner et al. 1978), and thyroidectomy of sexually immature rats has been shown to delay vaginal opening and to result in smaller ovaries, as well as uteri and vaginas that are not well developed (reviewed by Doufas and Mastorakos 2000). In porcine granulosa cell culture, follicle-stimulating hormone (FSH) and thyroid hormone act synergistically to stimulate granulosa cell differentiation and function (Maruo et al. 1987). Because of the interplay between the hypothalamic–pituitary–thyroid and the hypothalamic–pituitary–ovarian axes, we designed this study to evaluate the effects of low doses of 2,2′,4,4′-tetra-bromodiethyl ether (PBDE-47) on the developing reproductive system of the female rat. The results from the male studies will be presented elsewhere. PBDE-47 is one of the predominant congeners found in humans; Johnson-Restrepo et al. (2005) reported PBDE-47 concentrations of 1.3–2,700 μg/kg lipid in human adipose tissues samples collected in New York City. We treated pregnant rats to a single dose of PBDE-47 at 140 or 700 μg/kg body weight (bw) on gestation day (GD) 6. Assuming 20% of the body weight is composed of fat and 100% absorption of the compound, the experimental doses correspond to approximately 700 and 3,500 μg PBDE-47/kg lipid, respectively, which is well within or just above the range reported for humans. An additional group was treated with a low dose of the goitrogen 6-n-propyl-2-thiouracil (PTU) to serve as a reference for effects possibly associated with early developmental reductions in T_4_ and to ensure that our animal model is susceptible to thyroid hormone disruption. PTU inhibits thyroid peroxidase, thereby preventing the conversion of iodide to iodine and its incorporation into thyroglobulin. Inhibition of extra-thyroidal conversion of thyroxin to thyronine is also attributed to this compound (Knepel 2005).

## Materials and Methods

### Animals and housing

Virgin female Wistar rats (HsdCpb:WU; Fa. Harlan-Winkelmann, Borchen, Germany) weighing 200 ± 15 g were allowed to acclimate in our facility for 2 weeks. The rats were housed at a temperature of 21 ± 1°C and 50 ± 5% relative humidity with constant light/dark periods of 12 hr each. Tap water and rodent chow (Altromin 1324; Altromin GmbH, Lage, Germany) were given *ad libitum*. Two females were placed with one male for 3 hr on 8 consecutive days. Daily vaginal smears were examined for the presence of sperm. The day of sperm detection was considered day 0 of gestation. The pregnant females were housed in Type III macrolon cages with stainless steel covers and wood shavings (Altromin GmbH). The animals were treated humanely, and care was taken to ease suffering. The experimental protocol was approved by the Berlin Agency for Health and Social Welfare in accordance with the German National Animal Protection Law (Tierschutzgesetz 1998).

### Treatment

Three groups of females with sperm-positive vaginal smears were administered either pharmacologic grade peanut oil (Henry Lamotte GmbH, Bremen, Germany) as vehicle or PBDE-47 at 140 or 700 μg/kg bw (2,2′,4,4′-tetrabromodiphenyl ether, 98% purity; LGC Promochem GmbH, Wesel, Germany) by gavage (10 mL/kg bw) on GD6. An additional group, serving as reference control, was administered PTU (Sigma-Aldrich Chemicals GmbH, Steinheim, Germany). The gravid dams were given 5 mg/L PTU in the drinking water GD7 through postnatal day (PND) 21.

### End points

The number of litters was recorded for each end point. Eight dams from each group were sacrificed 27 days postpartum, and the ovaries were weighed and evaluated using light microscopy. The F_1_ offspring were weaned on PND22 and sacrificed on PND38; organ weights were recorded, and samples were either frozen at −80°C for measurement of aromatase activity or placed in Bouin fixative for histology. Trunk blood was collected, and the obtained serum samples were frozen at −20°C for measurement of circulating estradiol concentrations. A second set of female offspring was necropsied during estrus (based on vaginal cytology) on approximately PND100. We recorded body and organ weights and performed histologic evaluation of the ovary, uterus, vagina, and thyroid. At approximately 22 weeks of age, 22–24 virgin female offspring (F_1_) from each group were mated with non-exposed males to generate F_2_ offspring, so we could evaluate fertility and perform teratologic examinations of the skeletons.

### Ovarian follicle counting (PND38)

Whole ovaries (*n* = 9–10) were fixed in Bouins solution, dehydrated in ethanol, and embedded in paraffin; serial sections were cut every 6 μm and stained with hematoxylin and eosin (H&E). Primordial and primary follicles were counted in five sections per ovary, with the five sections taken from the middle of the ovary 240 μm apart. We counted only follicles in which the nucleolus could be seen. Secondary, tertiary, and atretic follicles were counted in 25 sections per ovary, with sections taken from the middle of the ovary 60 μm apart. Classification of ovarian follicles has been described in more detail (Talsness et al. 2005) and is based on a modification (Plowchalk et al. 1993) of a published scheme (Pedersen and Peters 1968); atretic follicles were identified by characteristics previously described by Borgeest et al. (2002) and Devine et al. (2000).

### Ovarian aromatase activity

We measured aromatase activity in ovarian homogenate according to the method of Hany et al. (1999). This involved detecting the aromatization of the A-ring of [1β-^3^H]androstenedione catalyzed by aromatase, which results in loss of tritiated hydrogen from the C-1β position and its integration into ^3^H_2_O. Ovaries were homogenized at a ratio of 1 mg tissue to 200 μL TEKS buffer (50 mM Tris HCl, 1 mM disodium EDTA, 100 mM potassium chloride, and 0.2 mM sodium azide, pH 7.4); the charged microtiter plate was incubated in a thermomixer at 37°C for 30 min before the reaction was stopped. The aromatase activity is expressed as femtomoles per milligram of protein per 15 min.

### Serum estradiol concentration

Trunk blood was collected at necropsy and allowed to clot on ice before centrifugation at 4°C for 15 min. The serum was collected and stored at −20°C until analysis. We measured the estradiol concentration in serum samples using a competitive radioimmunoassay kit according to the manufacturer’s instructions (Diagnostic Products Corporation, Biermann GmbH, Bad Nauheim, Germany). Counts per minute were detected and data were interpolated with a Cobra Auto-Gamma Counting System (Packard Instrument Company, Meridien, CT, USA).

### Light microscopy

We collected the ovaries (*n* = 4) from dams 28 days postparturition, and the ovaries (*n* = 4–6), uteri (*n* = 10–12), vaginas (*n* = 6–7), and thyroids (*n* = 10–12) from F_1_ female offspring (approximately 100 days of age) during estrus. All tissues were fixed in Bouins solution, dehydrated in ethanol, and embedded in paraffin. Sections (5 μm thick for the thyroid and 3 μm for all other tissues) were stained with H&E.

### Thyroid morphometry

We analyzed H&E-stained sections (5-μm) of the thyroid gland by standard point counting (Cruz-Orive and Weibel 1990; Serakides et al. 1999) to determine the proportions of colloid, follicular epithelium, and stroma. Photomicrographs of 10 fields per thyroid were taken at 200× magnification using a Zeiss Axiphot light microscope (Zeiss, Oberkochen, Germany) fitted with a Sony 3CCD camera (AVT Horn, Aalen, Germany). A grid with 300 intersections (points) was superimposed on each field; one of the three structural components under each intersection was identified and counted, giving a total of 3,000 points per animal.

### Electron microscopy

Tangential sections were made in the ovary and thyroid gland (*n* = 3) using a razor blade. Subsequently, the ovaries were cut crosswise for preparation of ultrathin sections. All samples were fixed in 1% glutaraldehyde plus 1% tannic acid in 0.1 M phosphate buffer (pH 7.4) and post-fixed in 1% osmium tetroxide in phosphate buffer. After rinsing and dehydration in an ascending alcohol series, the samples were embedded in Epon (Plano, Marburg, Germany), cut on a Reichert Ultracut microtome (Leica, Nussloch, Germany) followed by contrasting with 2% uranyl acetate/lead citrate. We evaluated the sections using an EM 10 transmission electron microscope (Zeiss).

### Female reproductive performance

At approximately 22 weeks of age, 22–24 female F_1_ offspring from each group were mated daily with untreated males for 14 days or until a sperm-positive vaginal smear was obtained.

On day 21 of gestation, the dams were sacrificed and the uterus was excised. We determined fetal weight and sex, as well as the numbers of implantations, resorptions, and fetuses. The fetuses were examined for external anomalies; all were cleared for skeletal staining by fixation in 5% formalin for 1 week and then rinsing in water for 2 days. After evisceration, they were placed in a diethylether/ethanol solution (1:4) for 1 week and then washed with water. The skeletons were stained with an alizarin/10% potassium hydroxide solution, rinsed with water, placed in a benzyl alcohol/glycerol/ethanol (1:2:2) solution until clear, and then stored in glycerol until examination.

### Statistical analyses

We performed statistical analyses using GraphPad Prism, Version 3, software (GraphPad Software Inc., San Diego, CA, USA). We considered the litter as the experimental unit. We compared means from the PTU group with those of controls using the unpaired Student’s *t*-test; means from the PBDE-47 groups were compared by analysis of variance (ANOVA) followed by the Dunnett’s test. Medians from the PTU group were compared with those of controls using the Mann-Whitney test, and those from the PBDE-47 group were analyzed with the Kruskal-Wallis test and Dunn’s Multiple Comparison Test. The ovarian weights of the dams and the organ weights of the offspring on PND38 were analyzed by analysis of covariance (ANCOVA) (SAS, version 9.1; SAS Institute Inc., Cary, NC, USA) using body weight and treatment as covariables, because statistically significant differences in body weights were ascertained for the PTU group compared with the control group.

## Results

### Body and ovarian weights of dams

At 27 days postparturition, the dams in the PTU group were heavier than those in the control group (*p* < 0.05). In the 140-μg PBDE-47 group, there was an increase in mean paired ovarian weight (*p* < 0.01) (Table 1).

### Ovarian histology of dams

We detected no histologic abnormalities in the ovaries (*n* = 4/group) of the dams from the control group or those exposed to 140 μg PBDE-47/kg bw. One of four animals in the 700-μg PBDE-47 group exhibited slight follicular dilation indicative of cysts. In the ovary from one animal in the PTU group, we observed expanded interstitial spaces, which is compatible with slight edema (not shown).

### Body and organ weights of F_1_ female off-spring on PND38

The mean body weight was significantly lower in offspring exposed to PTU than in controls on PND38 (Table 2). This is in contrast to PND1, when there was no statistically significant difference for any of the groups in average pup weight (whole litter body weight divided by the number of pups in the litter). Liver weight was significantly lower in both PBDE-47 groups. Paired ovarian weights were reduced in the 140-μg PBDE-47 group and those exposed to PTU. The change in ovarian weight was not associated with histopathologic alterations at the light microscopic level. Qualitative assessment revealed a decrease in tertiary follicles in the PTU and 700 μg PBDE-47 groups.

### Ovarian follicle numbers, ovarian aromatase activity, and serum estradiol concentration

**Figure 1:**
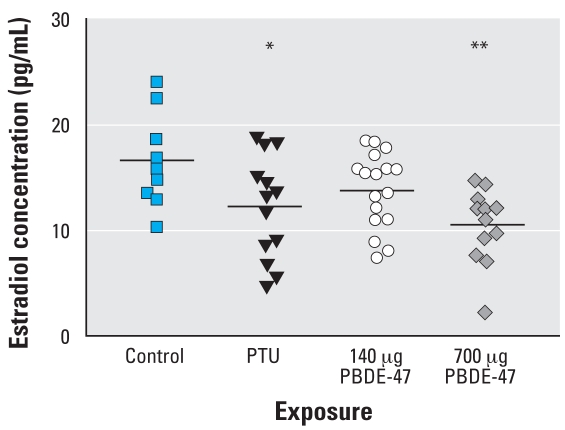
Individual serum estradiol concentrations (bars indicate means) of F_1_ female offspring on PND38 after treatment with vehicle or PBDE-47 (140 or 700 μg/kg bw) to F_0_ dams on GD6. PTU was administered on GD7–PND21. *p < 0.05, and **p < 0.01.

**Figure 2:**
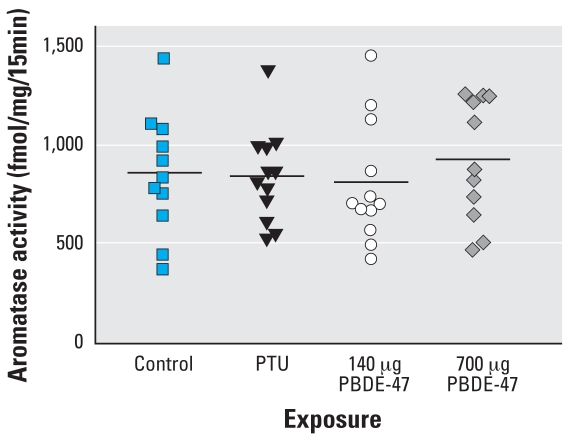
Individual ovarian aromatase activity (bars indicate means) of F_1_ female offspring on PND38 after treatment with vehicle or PBDE-47 (140 or 700 μg/kg bw) to F_0_ dams on GD6. PTU was administered on GD7–PND21.

We found statistically significant differences in follicle numbers in the PTU and 700-μg PBDE-47 groups: primordial and tertiary follicles were reduced in the PTU group, and reductions in secondary and tertiary follicles occurred in the PBDE-47 group (Table 3). The reduction in growing follicles in the 140-μg PBDE-47 group did not reach statistical significance. The serum estradiol concentrations were reduced in the treatment groups, and were statistically significant in the PTU and 700-μg PBDE-47 groups (Figure 1). Whole ovarian aromatase activity was similar to control in all treatment groups (Figure 2).

### Body and organ weights of female offspring in estrus on PND100

We observed no differences in body weight or reproductive organ weights in the treatment groups compared with controls. The only statistically significant changes were a reduction in liver weight and an increase in thyroid weight in the PTU group (Table 4).

### Histology of F_1_ female offspring on PND100

**Figure 3:**
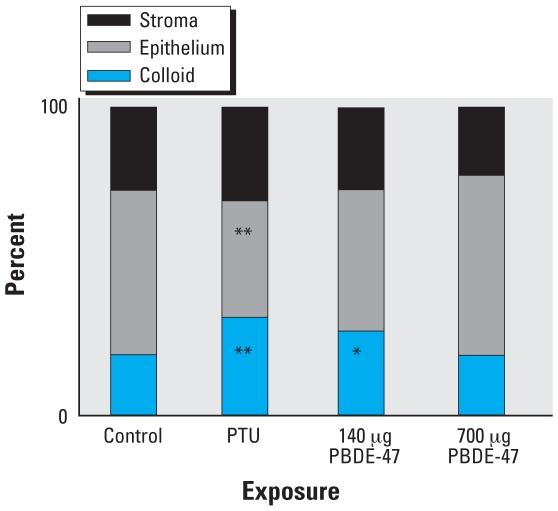
Percent of intersections overlying either stroma, epithelium, or colloid of thyroid glands from F_1_ female offspring on approximately PND100 after treatment with vehicle or PBDE-47 (140 or 700 μg/kg bw) to F_0_ dams on GD6. PTU was administered on GD7–PND21. The control group includes 9 litters; the PTU and PBDE groups (140 and 700 μg/kg) include 10 litters each. *p < 0.05, and **p < 0.01.

**Figure 4:**
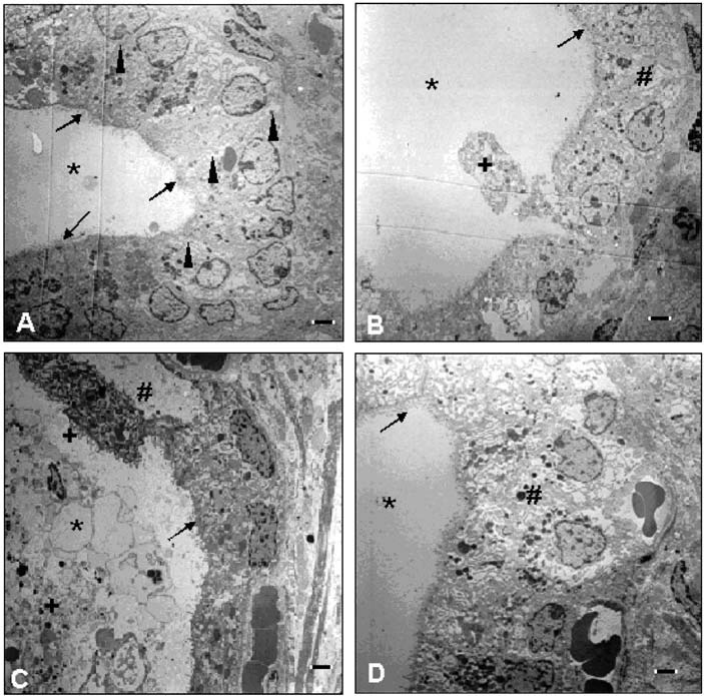
Electron micrographs showing ultrastructure of thyroid from F_1_ female offspring on approximately PND100 after treatment with vehicle or PBDE-47 (140 or 700 μg/kg bw) to F_0_ dams on GD6. PTU was administered on GD7–PND21. (*A*) Thyroid section from control animal. The follicular architecture consists of a single layer of thyrocytes, with adjacent cells in close contact (arrowheads) surrounding a colloid-filled lumen (*). Microvilli are present on the luminal side (arrows) of the polarized thyrocytes. In sections from animals exposed to PTU (*B*), 140 μg PBDE-47/kg (*C*), and 700 μg PBDE-47/kg (*D*), the follicles have an irregular, nontypical shape. Numerous follicular cells are detached (+) from the basal membrane, and the follicle cells are swollen and dilatated (#). Magnification = 5,000×; bar = 1 μm.

At the light microscopic level, the histologic findings of the ovary, uterus, and vagina were unremarkable compared with controls. Evaluation of the thyroid glands revealed occasional follicular cyst formation in the 140-μg PBDE-47 and PTU groups, and only mild cyst formation in the 700-μg PBDE-47 group. There were multiple areas of degenerated follicular epithelium in the 140-μg PBDE-47 group and slight attenuation of the follicular epithelium in the PTU group. Morphometric analyses resulted in compatible results, as thyroid point counting yielded a statistically significant decrease in the number of points overlying the follicular epithelium in the PTU group, as well as an increased number over the colloid in the PTU and 140-μg PBDE-47 groups (Figure 3). The number of points overlying the epithelium in the 140-μg PBDE-47 group was decreased and, although statistical analysis indicated exposure-related differences, the post hoc test for this end point but did not reach statistical significance. Electron microscopy also revealed detachment of thyroid follicular epithelial cells, which can be found in the colloid (Figure 4)

**Figure 5:**
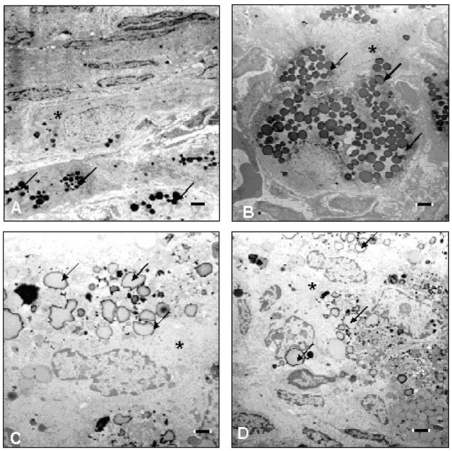
Electron micrographs showing ultrastructure of ovaries from F_1_ female offspring in estrus on approximately PND100 after treatment with vehicle or PBDE-47 (140 or 700 μg/kg bw) to F_0_ dams on GD6. PTU was administered on GD7–PND21. (*A*) Control ovary showing vesicular structures with homogeneously dense granular material in the cytoplasm (*) of the stromal cells (arrows). (*B*) In PTU-exposed ovary, there is an increase in vesicular structures with homogeneously dense granular material (arrows) in the cytoplasm (*). Multiple vacuolization and large vesicles with homogeneously dense granular material (arrows) in the cytoplasm (*) of ovarian cells are present in the ovaries from 140 μg PBDE-47 (*C*) and 700 μg PBDE-47 (*D*) ovaries. Magnification = 5,000×; bar = 1 μm.

Ultrastructural analysis of the control ovaries revealed the presence of intact stromal cells with a small number of vesicular structures and a few vacuoles containing small electron dense granular masses (Figure 5A). The stromal cells of the ovary from the PTU-treated group (Figure 5B) have an accumulation of vesicular structures with homogeneously dense granular material. The ovaries from animals treated with 140 (Figure 5C) and 700 μg PBDE-47/kg (Figure 5D) showed an accumulation of vesicular structures with homogeneously dense granular material in the cytoplasm of the stromal cells, which appear to fuse together to form large vacuoles.

### Reproductive performance and teratology

We found no differences between the control group and any of the treatment groups in terms of the number of live fetuses, fetal weight, or resorption rate. The mean number of implantation sites per dam was significantly increased in the PTU group. The sex ratio of the F_2_ animals in the 700-μg PBDE-47 group was approximately one–half that of the control group (Table 5). However, comparison of the altered sex ratio with controls from two different historical experiments (*n* = 24 and 43 litters) revealed no differences.

Evaluation of the F_2_ offspring from the F_1_ female offspring mated with untreated males revealed two anomalies in one pup (F_2_) from the 700-μg PBDE-47 group: a shortened mandible accompanied by fused tympanic bone.

## Discussion

PBDEs have been shown to alter thyroid hormone homeostasis, and interactions have been reported between thyroid hormones and the reproductive system. We evaluated the influence of early developmental exposure to PBDE-47 on the female reproductive system.

The increase in ovarian weight observed in the dams at the low PBDE dose (140 μg/kg) was not observed in the group exposed to the higher dose of PBDE-47 (700 μg/kg). Characterization of the dose–response relationship was not possible in this study; however, there are reports in the literature describing nonmonotonic dose–response curves after exposures to hormonally active compounds (Almstrup et al. 2002; Muto et al. 2002; Putz et al. 2001; vom Saal et al. 1997), indicating that qualitative differences can exist between low and high doses. Possible mechanisms include differential binding affinities of compounds to steroid receptor isoforms, competition between endogenous and exogenous ligands, and the formation of mixed ligand–receptor complexes versus homodimers and their respective recruitment of activators or repressors of gene transcription. In addition, nongenomic effects of steroids may modify genomic actions yielding nonmonotonic responses (Rochette-Egly 2003).

On PND38, we found a reduction in body weight and paired ovarian weight in the group exposed to PTU, which is in accordance with another study in rats after oral PTU treatment from PND21 to PND40 (Marty et al. 1999) and one after exposure from PND1 to PND40 (Dijkstra et al. 1996). In the present study, we also found reduced ovarian weight in the 140-μg PBDE-47 group. This reduction is in contrast with the increase in ovarian weight in the mothers from the same treatment group and it was also not associated with histologic abnormalities at the light microscopic level.

We observed statistically significant alterations in folliculogenesis in offspring in the PTU group and the 700 μg PBDE-47 group. PTU exposure resulted in a 50% reduction in primordial follicles, posing the possibility that these animals may experience early sexual senescence. (The disadvantages of early menopause in humans include a shorter reproductive life span; also, the onset of menopause can be associated with a variety of health problems such as osteoporosis.) The tertiary follicles were also reduced following exposure to PTU. Modifications to folliculogenesis have been reported in other studies after exposure to PTU (Chan and Ng 1995; Dijkstra et al. 1996), and these data are in agreement with a study performed with ammonium perchlorate (AP), which is used to treat hyperthyroidism and is also found as a water contaminant in the United States because of its use in rocket fuel, paints, fertilizers, and lubricants. Baldridge et al. (2004) reported a reduction in preantral follicles, as well as total antral follicles, following *in utero* and lactational exposures to high doses of AP, whereas lower doses affected only the large antral follicles.

In the present study, exposure to PBDE-47 did not affect primordial follicles as in the PTU group; however, a similar effect on larger follicles was demonstrated, as secondary and tertiary follicles were decreased in the 700-μg PBDE-47 group. The lower number of larger follicles in the PTU and PBDE-47 groups was not due to an increased rate in atresia of this follicle stage. PCB mixtures (Baldridge et al. 2003; Lilienthal et al. 2006) and high doses of PBDE-99 (Lilienthal et al. 2006) have also been shown to alter folliculogenesis. Ovarian folliculogenesis was not altered, however, during adulthood following prenatal and lactational exposure to low doses of PBDE-99 (Talsness et al. 2005).

In the studies by Baldridge et al. (2003, 2004), T_4_ supplementation was able to ameliorate the effects on the smaller sized follicles, suggesting that thyroid hormone disruption plays a role in the disturbed folliculogenesis of the less mature follicles.

Antral follicles are a major source of estrogen, and we observed a concomitant reduction in circulating estradiol concentrations after exposure to either PTU or PBDE-47. In a study following *in utero* exposure to 1 or 10 mg PBDE-99/kg, Lilienthal et al. (2006) reported effects on circulating estradiol concentrations. They observed statistically non-significant reductions in circulating estradiol concentrations, which were more pronounced in the lower dose group than the higher one, in F_1_ females on PND21. Estradiol concentrations in males, however, were decreased in a statistically significant fashion on PNDs 21 and 160 (Lilienthal et al. 2006). Evidence suggests that some PBDE congeners and metabolites may affect CYP19 activity. Cantón et al. (2005) reported that aromatase (CYP19) activity evaluated in the H295R human adrenocortical carcinoma cell line showed inhibition of aromatase activity with 6CH_3_O-PBDE-47. However, in the present study, we found no changes in whole-ovary aromatase activity associated with reduced circulating estradiol concentrations. Some explanations for the decreased estradiol concentrations include the lower number of antral follicles, altered gonadotropins affecting follicular maturation, and the expression of steroidogenic enzymes other than aromatase or an increase in estrogen metabolism.

Tonic levels of FSH play a role in early follicular growth, and rising FSH levels are involved in further follicular maturation when expression of steroidogenic enzymes increases dramatically. The alterations in folliculogenesis and steroidogenesis indicate disruption along the hypothalamic–pituitary–ovarian axis.

At necropsy during estrus on approximately PND100 of the present study, the reduction in body weight the PTU-exposed offspring observed on PND38 was no longer apparent. Persistent adverse effects on the thyroid gland after exposure to PTU was indicated by increased weight of the thyroid gland associated with histologic changes, indicating occasional follicular cyst formation and attenuation of the follicular epithelium. Although no change in thyroid weight was apparent in the animals exposed to 140 μg PBDE-47/kg, we observed similar histologic findings. Thyroid point counting, performed by an observer unaware of the histologist’s findings, supported the histologic observations in the PTU group: the proportion of points over the epithelium were decreased, and the number over the colloid were increased in the this group. The same pattern was observed in the morphometric analysis of the 140 μg PBDE-47 group; however, the decrease in the epithelium did not reach statistical significance. Developmental exposure to either PTU or PBDE-47 led to changes in the thyroid tissue, which were apparent in adulthood.

At adulthood, the increased amount of vesicles observed in the ovaries from the offspring exposed to PTU or PBDE-47 exhibited ultrastructural changes similar to those we reported following exposure to PBDE-99 (Talsness et al. 2005). This observation is compatible with nonspecific or uncontrolled synthesis of steroid products.

The mean increased number of implantation sites in the PTU-exposed F_1_ females was accompanied by a higher resorption rate, resulting in a similar mean number of fetuses compared with the control group. The higher resorption rate in this group is not considered to be biologically significant because resorption rates of ≤10% are within normal limits for our historical controls of this rat strain. We observed a statistically significant alteration in the secondary sex ratio in favor of females after mating of the F_1_ females from the 700-μg PBDE-47 group. The biological relevance of this finding is low because analyses performed comparing the 700-μg PBDE-47 group with our historical controls indicated no statistically significant differences.

The anomaly observed in one F_2_ offspring following exposure of the F_0_ dam to 700 μg PBDE-47/kg on GD6 is one that we have never observed in our rat strain after examining > 10,100 fetuses (historical data). In a similar experiment, we also observed skeletal anomalies in offspring from two different mothers exposed *in utero* and via lactation to 300 μg PBDE-99/kg, which had also never been documented in our laboratory (Talsness et al. 2005). Incomplete bone deposition was observed in the left and right parietal and frontal bones of the skull in one offspring. Also, in a pup from another litter of the same group, only a portion of the first sacral vertebra was present and the remaining sacral and caudal vertebrae were absent.

Possible causes for these anomalies may be either spontaneous or substance related. The fact that we have not observed these anomalies in Wistar rats speaks against a spontaneous cause, although it cannot be ruled out. In addition, the F_0_ generation was treated with a very low dose of PBDE-47, and the anomaly was seen in the F_2_ generation; this suggests that the anomaly is not directly substance induced, as the congener was probably not present at the time of mating. It is theoretically possible that it is related to an epigenetic modification of the DNA.

## Summary and Conclusions

Data from the present study indicate endocrine disruption following *in utero* and lactational exposure to environmentally relevant doses of PBDE-47, as the doses used in this study would result in an approximate maternal body burden within or just above the range of concentrations reported in human adipose tissue samples collected in New York City (Johnson-Restrepo et al. 2005). We observed alterations in ovarian folliculogenesis, circulating estradiol concentrations, and persistent changes to both the ovaries and thyroid glands. Legislation banning the marketing and use of the pentaBDE and octaBDE commercial formulations in the European Union and some states of the United States has already occurred, and decaBDE has been banned in Sweden and in Washington and Maine; however, these lipophilic compounds are highly persistent in the environment, and release and exposure will continue for an extended period of time. The European Union is considering a vote to discontinue the planned ban of the decaBDE formulations. The continued use of decaBDE is of concern because of direct exposure to the compound and its debromination to lower brominated congeners. In addition, exposure to the myriad of chemicals in the environment yields the possibility of additive, synergistic, or antagonistic effects. The developing embryo, fetus, and neonate are highly susceptible to exogenous insults, and the magnitude of the current maternal body burden of PBDEs may be of concern for human health.

## Figures and Tables

**Table 1:** Body weight and paired ovarian weight of dams (F_0_) 27 days postparturition.

Treatment	Body weight (g)	Paired ovaries (mg)
Control	232 ± 6	92 ± 4
PTU	248 ± 18*	101 ± 4
140 μg PBDE-47/kg	237 ± 16	108 ± 4**
700 μg PBDE-47/kg	232 ± 7	91 ± 4

*n* = 8 per group. Body weights are presented as mean ± SD, and paired ovary weights are mean ± SE adjusted for body weight.

* *p* < 0.05, and

** *p* < 0.01 by ANCOVA.

**Table 2:** Body weight and selected organ weights of F_1_ females on PND38.

Treatment	Body weight (g)	Liver (g)	Uterus (mg)	Paired ovaries (mg)
Control (*n* = 11)	44 ± 7	2.00 ± 0.06	24 ± 2	23 ± 1
PTU (*n* = 15)	38 ± 4**	1.86 ± 0.06	25 ± 2	15 ± 1**
140 μg PBDE-47/kg (*n* = 18)	42 ± 6	1.80 ± 0.05*	24 ± 2	17 ± 1**
700 μg PBDE-47/kg (*n* = 16)	45 ± 4	1.73 ± 0.06**	25 ± 2	21 ± 1

*n* indicates the number of litters. Body weights are presented as mean ± SD (by ANOVA and unpaired *t*-test). Organ weights are presented as mean ± SE adjusted for body weight (by ANCOVA).

* *p* < 0.05, and

** *p* < 0.01.

**Table 3:** Ovarian follicle counts for F_1_ females on PND38.

	Follicle type				
Treatment	Primordial	Primary	Secondary	Tertiary	Atretic
Control (*n* = 9)	78 (62, 100)	46 (35, 50)	7 (7, 8)	13 (9, 16)	41 (35, 46)
PTU	42 (28, 76)*	32 (18, 43)#	5 (2, 9)	9 (4, 12)*	36 (26, 46)
140 μg PBDE-47/kg	76 (56, 93)	35 (32, 46)	4 (4, 8)**	11 (5, 14)	40 (36, 52)
700 μg PBDE-47/kg	82 (69, 105)	42 (28, 50)	4 (2, 7)*	8 (4, 10)*	45 (29, 56)

The median number of follicles and (Q_1_, Q_3_) presented are from 5 sections per ovary for the primordial and primary follicles and from 25 sections per ovary for the secondary, tertiary, and atretic follicles. The control group includes 9 litters; the PTU and PBDE groups (140 μg/kg and 700 μg/kg) include 10 litters each.

* *p* < 0.05,

** *p* = 0.06, and

# = 0.08 by Kruskall-Wallis test, followed by Dunn’s Multiple Comparison Test and Mann-Whitney test).

**Table 4:** Body weight and selected organ weights of the F_1_ females in estrus on approximately PND100.

Treatment	Body weight (g)	Liver (g)	Thyroid (mg)	Uterus (mg)	Paired ovaries (mg)
Control (*n* = 14)	167 ± 13	6.89 ± 0.52	10 ± 2	470 ± 53	98 ± 9
PTU (*n* = 16)	168 ± 16	6.28 ± 0.80*	12 ± 2** (*n* = 15)^a^	480 ± 56	100 ± 12
140 μg PBDE-47/kg (*n* = 19)	174 ± 26	7.02 ± 0.74	10 ± 1	461 ± 58	98 ± 12
700 μg PBDE-47/kg (*n* = 18)	162 ± 14	6.60 ± 0.61	11 ± 2 (*n* = 16)^a^	464 ± 123	103 ± 13 (*n* = 17)^a^

*n* indicates the number of litters. Body and organ weights are presented as mean ± SD (by ANOVA, followed by Dunnett’s Test and unpaired *t*-test).

a Number of litters differs from that given for the treatment group.

* *p* < 0.05, and

** *p* < 0.01.

**Table 5:** Fertility indices of F_1_ female offspring after mating with nonexposed males.

	Total no. of implantation sites	Total no. of live fetuses	Implantation sites per dam (mean ± SD)	Fetuses per dam (mean ± SD)	Mean fetal weight (g) (mean ± SD)	Resorption rate (%)	Sex ratio [median (Q_1_,Q_3_)]
Control (*n* = 11)	133	125	12.1 ± 0.8	11.4 ± 0.8	4.7 ± 0.2	6	1.20 (0.84, 2.38)
PTU (*n* = 17)	223	200	13.1 ± 1.2*	11.8 ± 1.8	4.7 ± 0.2	10	1.17 (0.71, 1.45)
140 μg PBDE-47 (*n* = 19)	243	228	12.8 ± 1.6	12.0 ± 2.0	4.7 ± 0.5	6	0.86 (0.53, 1.42)
700 μg PBDE-47 (*n* = 17)	212	202	12.5 ± 1.1	11.9 ± 1.4	4.5 ± 0.3	5	0.65 (0.45, 1.12)*

*n* = number of litters. Sex ratio is calculated as male/female. Analyzed by ANOVA followed by Dunnet’s test; unpaired *t*-test; Kruskal-Wallis test followed by Dunn’s; and Mann-Whitney test.

* *p* < 0.05.
